# Decision making on (dis)continuation of long-term treatment in mental health services is an interpersonal negotiation rather than an objective process: qualitative study

**DOI:** 10.1186/s12888-019-2072-0

**Published:** 2019-03-18

**Authors:** B. Koekkoek, B. van Meijel, A. Perquin, G. Hutschemaekers

**Affiliations:** 1 HAN University of Applied Sciences, Research Group Social Psychiatry & Mental Health Nursing, Kapittelweg 33, 6525 EJ Nijmegen, The Netherlands; 2 Inholland University of Applied Sciences, Amsterdam, The Netherlands; 3 Department of Psychiatry, Amsterdam Public Health research institute, Amsterdam UMC, VU Amsterdam, Amsterdam, The Netherlands; 4 Parnassia Psychiatric Institute, The Hague, The Netherlands; 5 GGZ-VS, Academy for Masters in Advanced Nursing Practice, Utrecht, The Netherlands; 6 Research Group Clinical Psychology, Radboud University, Nijmegen, The Netherlands; 7 Pro Persona Mental Health Services, Wolfheze, The Netherlands

**Keywords:** Netherlands, Mental disorders, Mental health services, Psychosocial treatment, Therapy, Grounded theory, Qualitative research, Allocation of treatment resources, Decision making

## Abstract

**Background:**

Research into termination of long-term psychosocial treatment of mental disorders is scarce. Yearly 25% of people in Dutch mental health services receive long-term treatment. They account for many people, contacts, and costs. Although relevant in different health care systems, (dis)continuation is particularly problematic under universal health care coverage when secondary services lack a fixed (financially determined) endpoint. Substantial, unaccounted, differences in treatment duration exist between services. Understanding of underlying decisional processes may result in improved decision making, efficient allocation of scarce resources, and more personalized treatment.

**Methods:**

A qualitative study design, according to Grounded Theory principles, was used to understand the decision making process. In four teams in three large Dutch mental health services, 29 multidisciplinary case conferences were observed, and 12 semi-structured interviews were conducted.

**Results:**

We describe two constituent elements of decision making: the process through which decision making is prepared and executed, and the substantial factors guiding its outcomes. The first consists of: (1) steps towards a team discussion on treatment termination, (2) team-related factors that influence decisions, and (3) the actual team decision making process. The second consists of factors related to patients, professionals, organization, and wider environment. Our main finding was that discussions of treatment (dis)continuation are highly unstructured. Professionals find it difficult to discuss with patients and teams, team discussion are ad-hoc, and clear decisions are scarce. We offer four explanations: first, long-term treatment lacks golden rules on outcome and process to base decisions on. Second, in the absence of such rules professionals rely on experience but underappreciate their own biases. Third, consequently, professionals aim for decisional consensus, which however is scarce among professionals. Fourth, treatment environments are hardly in favour of changing default (continuation) settings.

**Conclusion:**

Clear decision making, and terminating treatment when appropriate, is systematically hampered within secondary mental health services. Since continuation is the ‘easy’ default option, discontinuation requires skillful and determined navigation of interpersonal negotiations. Given services’ scarce means, people’s large demands for help, and patients’ unused potential autonomy, it is desirable to invest in decision making skills and procedures – both human and economic benefits may be substantial.

## Background

Research into the termination of long-term treatment of mental disorders is nearly absent [1, 2]. While short-term, protocol-based psychotherapy has been subject of rigorous trials [3], treatment lasting longer than 20–30 sessions or a year is largely unmonitored [4]. There is a substantial, non-empirical, literature on terminating long-term analytic psychotherapy [5], but not on long-term psychosocial treatment. Psychosocial treatment is a broad term for talking therapy aimed particularly at support and recovery, and less at change and cure. It includes treatment as provided by community mental health teams in the Netherlands, the UK and the US [6] and is generally offered by secondary mental health services. Psychosocial treatment, being cheaper and more easily accessible to people with complex problems, is far more frequently applied than psychotherapy while it faces equally scarce resources. Timely termination is necessary to maintain availability of services for other people seeking treatment [7], and to avoid patients’ unnecessary dependency on services [8, 9].

Outcomes of psychotherapy-trials for common mental disorders are unequivocal: optimal length of treatment ranges between 10 and 13 sessions, largest effects are observed at the start, and early drop-outs are more often found with less experienced therapists, and among certain patient groups (e.g. people with personality disorders, younger people, and people with more serious disorders) [10, 11]. In contrast to research settings, modal treatment duration in general mental health practice is 3–5 sessions [11]. While some 6% of the Dutch yearly receive some form of treatment for mental health problems, 25% receives psychosocial treatment for over a year [12]. Of this group, 13% receives many sessions, resulting in 40% of costs [12]. Apparently, once the respective limits of brief treatment have been crossed, formal criteria on treatment duration are unavailable [9]. Long-term psychosocial treatment may be expected for people with disorders traditionally seen as severe and ‘chronic’ (e.g. psychotic and bipolar disorders). Yet, also people with other disorders – often seen as ‘common’ and thus less severe or chronic – do receive such long-term treatment [12, 13]. Thus, it is not only the type or perceived severity of a mental disorder that is relevant to treatment duration.

Scientific guidelines for decision making on (dis)continuation of long-term psychosocial treatment (if, when, and under which conditions) is absent. Mental health services, in the Netherlands as well as the UK, rarely provide clear guidelines to their employees. Financial guidelines for treatment duration exist through the Diagnosis Related Groups system (DRG), yet they may – under universal health coverage like the NHS or comparable schemes such as in the Netherlands – easily be bypassed through re-classifying, or re-indicating patients [12]. Economic restraints such as general budget cuts, which vary over time and place, may have enormous impact on services but in themselves do not offer guidelines on which treatment should be discontinued at what moment. While patient-related factors may play an important role, cross-service comparisons show major differences in psychosocial treatment duration and recovery rates. These can only be partly accounted for by case-mix differences – i.e. services treating different types of patients [14]. We hypothesize that, in the absence of unequivocal guidelines, other factors account for varying psychosocial treatment duration. These could include professionals’ feelings towards patients [15, 16], patients’ incentives to stop or continue treatment [16–18], patient-professional interactions [19], teams’ group dynamics [20, 21], and other variables shaping decision making on treatment (dis)continuation. Interpersonal processes and negotiations in and around mental health services, previously identified as relevant to for instance individual patient diagnoses [22], service arrangements [23], and professional decisions [24], are explored in this study.

Research into these interpersonal factors is needed to improve decision making on psychosocial treatment (dis)continuation, since ‘top-down’ national policies such as treatment and financial guidelines do not eliminate variation. This knowledge may result in more insight into the social processes that shape decisions, and possibly result in psychosocial treatment optimization, efficient allocation of scarce resources, and more personalized treatment and care. Our research question therefore is: on what grounds, and by which processes, do multidisciplinary outpatient teams, individual professionals, and clients decide to either terminate or continue psychosocial treatment which has lasted for more than a year (or 30 sessions)?

## Methods

### Study design

We used a qualitative study design, based on Grounded Theory principles [25, 26]. Grounded Theory was chosen since we aimed to understand decision making as a process, and to develop a micro-theory on how decisions are made and which factors influence this process. Since in large mental health services treatment decisions are generally made by multidisciplinary teams during regular case conferences, we observed such meetings in a variety of teams. These observations were complemented with individual interviews, in order to also obtain in-depth insight into the individual perspective of professionals and patients with respect to decision making.

### Setting and sample

Multidisciplinary teams within three large mental health services in the south east and central Netherlands were invited to participate in the study. These three services had, in previous informal contacts with the research group, voiced capacity problems due to large influx and limited outflow of people in need of services. A total of seven teams was informed about the study, stating its purpose as ‘a study into multidisciplinary team decision making on treatment termination’, involving participant observation and individual interviews. Three teams declined participation (due to ‘lack of time’), after having been given background information on previously discussed capacity problems as an incentive for the study. Declining teams matched teams that did participate concerning professionals employed and patients targeted.

The actual sample consisted of four teams, each with a specific target population (i.e. mood disorders, personality disorders [two teams], and severe and long-term substance use disorders). The latter team offered time-unlimited assertive outreach community treatment, the other three provided regular (office based) outpatient treatment with a maximum duration of one to 2 years. These teams were selected because of (1) the variation in target groups and treatment type, and (2) their willingness to allow a researcher in their case conferences. Since treatment duration of patients with personality disorders is long in the Netherlands [13] and treatment termination therefore apparently difficult, we selected two teams working with this group.

In total 29 multidisciplinary case conferences (MCCs) were observed, lasting between 30 and 120 min each. Attendance varied from 5 to 16 people. A high number of meetings (11) was observed in one particular team, due to early participation of this team in this study, and due to theoretical sampling choices (i.e. the occurrence of many events relevant to the research question). Also, 12 semi-structured individual interviews were conducted, lasting for 60–90 min. Individual professionals were selected through participating teams, based on (1) variation in professional background and working experience, and (2) their professional behaviour in team meetings (i.e. notably active or passive in reaching decisions on treatment termination). Individual patients were invited by these participating professionals. All professionals invited for an individual interview were willing to participate. Also, all of the invited patients who had recently ended care, or were going to end care within 2 months, participated. Professionals were invited informally by the researcher, following or preceding team meetings. Patients, after having responded positively to the professional’s invitation, were contacted by phone, informed of the study, and – if willing to participate – invited for a face-to-face meeting with the researcher. All individual interviews took place in the participating services, although patients were offered alternative locations (i.e. in a neutral setting, at their own house, or a location of their choice). During this meeting, oral and written information on the study was provided, and informed consent was asked.

### Data collection: Team observations

The decision-making process of teams was observed through attendance of multidisciplinary case conferences (MCCs), with no interference of the researcher: (1) all verbal communication was recorded on audiotape, (2) all relevant non-verbal information (e.g. the Diagnosis Related Group-status [DRG], of a case which was often visible on paper/ screen but not always verbally announced) was summarized in a notebook, (3) non-verbal team-interactions were also noted. The teams were aware of the researcher’s role and goals, but did not display any difficulties or restraints with it. The researcher had previous working experience in teams such as studied and therefore was able to blend in relatively easily [27]. If possible, immediately after the meeting, questions were asked to individual professionals to clarify uncertainties that remained after the conference (this data was also noted).

### Data collection: Individual interviews

In congruence with principles of Grounded Theory, interviews started with a broad opening question. For professionals, this was: ‘Can you tell me about how you decide to terminate or continue treatment and which factors influence this decision?’. For clients, this was: ‘Can you tell me about how and by whom the decision was made to terminate treatment, and which factors influenced this decision?’. Interviewees were encouraged to reply as comprehensively as possible to this opening question in the interview’s initial phase. Next, topics brought forward by the interviewee were explored in more detail. For interview support, a self-developed topic guide (based on the existing literature) was used to elicit details on decision making that were not spontaneously covered. Topics included: when treatment is ‘good enough’, how to assess this, how to discuss possible termination, how to discuss possible dissenting views between patient and professional, the influence of the professional, organisational, and financial context on this process, and others. The interviews took place at a location chosen by the interviewees, were audio recorded and transcribed verbatim.

### Data analysis

**Fig. 1:**
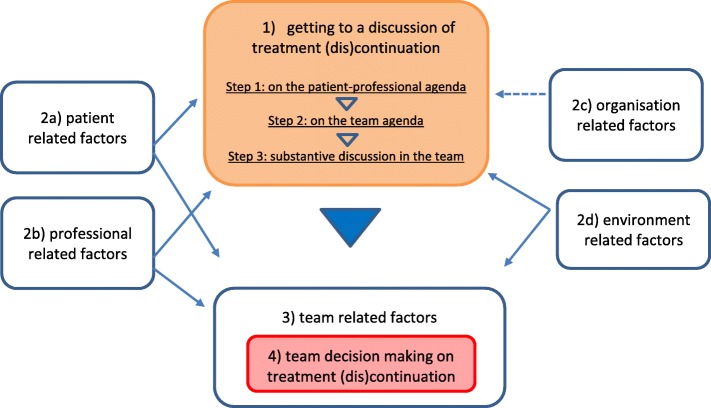
Overview of treatment termination decision making as an interpersonal negotiation

Data collection and analyses were conducted between September 2013 and January 2015, using ATLAS-Ti 7.0 software. Two members of the research team independently coded four interviews. Given the comprehensiveness of data the from team meetings, resulting in many possible codes and perspectives, we considered coding by two team member followed by discussion necessary for development of a consistent code tree for subsequent analysis. During the research process, while alternating data collection and intermediate analysis, data from previous observations and interviews were used to deepen understanding of the issues mentioned and facilitate ‘theory’ development. For instance: if a professional stated that financial factors explained treatment termination, the researcher explored why other professionals were able to continue treatment while facing equal financial constraints. We used open, axial, and selective coding in accordance with Grounded Theory. An example of an open code is for instance ‘not wanting to quit’, closely resembling the observed text, which was then categorized under ‘reluctance to end treatment’, then under ‘motivation’, and finally became part of the main category ‘patient-related factors’. However, since ‘reluctance to end treatment’ was also found in professionals, this code was also explored and coded in detail using professionals’ quotes. While doing this, we sought for instance in the data for causes and consequences of this reluctance (*axial coding*). When patient-related factors became part of the core process in this study (Fig. 1), this category was further explored in relation to other categories in the model (*selective coding*).

**Table 1:** Description of participating teams

	Team 1	Team 2	Team 3	Team 4
Mental health service	1	2	2	3
Target group (diagnosis)	Mood disorders	Personality disorders	Personality disorders	Long term substance use disorders
Treatment duration	1–2 years	1–2 years	1–2 years	unlimited
Team age range	29–60	30–64	40–60	30–63
Team size	9	10	7	15
Number of conferences observed	6	11	6	6
Team composition^a^				
Psychologist/psychotherapist	1	6	3	1
Psychiatrist	1	1	1	1
Physician/doctor	3	1		1
(Community) Mental health nurse/nurse practitioner	3	2	3	3
Social worker	1			5
Other (e.g. occupational therapist, expert by experience)				4

^a^team composition varied due to random variation (teams 1–3), and to type of treatment offered by an outreach team (team 4), usually consisting of more nurses, social workers and others, and fewer psychologists

The research team met each 6–8 weeks to discuss progress, with particular attention for the researcher’s objectiveness as an observer/analyser. Also, interview techniques and interpersonal style were monitored, intermediate analyses were evaluated and discussed – including consequences for further data collection and analysis. Emerging categories and their mutual relationships were selected and explored, and theoretical sampling strategies for following observations and interviews were designed. Theoretical sampling resulted mainly in (1) attending more meetings in one or two specific teams, and (2) inviting specific professionals for individual interviews.

Halfway through the observations and interviews, a preliminary overall model was constructed, existing of five large categories. Also, a tentative model with three successive stages in the decision making process was designed. A preliminary core category was identified (‘treatment (dis)continuation depends on interpersonal negotiation’). Finally, an extensive thick description of the study findings was written, with a comprehensive elaboration of the decision making processes placed within the specific contexts in which they occurred. The findings were structured according to the three stages and five descriptive categories. It was discussed and commented on in the research team, resulting in a number of additional questions used in the following observations and interviews to clarify, refine, and expand the categories and the theoretical model (Table 1).

## Results

In order to answer our research questions we describe two constituent elements of decision making in teams (Table 1): the process through which decision making is prepared and executed, and the substantial factors guiding the outcome of decision making. Since these two elements are interdependent, we present them as a scheme (Fig. 1), and in the following sub-sections describe them: (1) sequence of steps that need to be taken before a team discussion on treatment termination takes place, (2) substantial factors/grounds on which decisions may be made, (3) team-related factors on which team decisions may be made, and (4) the actual team decision making process.

1. **The process of getting to a discussion of treatment (dis)continuation**

Our main finding was that default often is to continue treatment without a clear consideration or discussion – unless either patient, professional or team explicitly initiated such discussion. However, before getting there, two previous conditional steps were required. First, patient and professional need to discuss treatment (dis)continuation in their contact. Second, the professional needs to bring up treatment (dis)continuation in the team.

### Getting treatment (dis)continuation on the patient-professional agenda

We found that treatment (dis)continuation often only became a topic if (1) the patient had a strong incentive to discuss it, (2) the professional had a strong incentive to do so, or (3) there were strong organisational incentives.

First, *patient incentives* were found to be twofold: 1) being reluctant to be in treatment, therefore wishing to end treatment, or 2) being satisfied with treatment and feeling recovered, therefore wishing to end treatment. The patients’ citations below express a certain discontentment with treatment or the mental health service.

Patient (Interview 3).

*I told my CMHN clearly: ‘In July I will quit. And I have also explained what is wrong with mental health care as I see it.*

Patient (Interview 11).

*Then I thought: this is enough. It* [treatment] *does not fit me anymore. Actually, I would stay until September because there would be some team evaluation and they wanted to use my story for it. I would have to stay longer for that, but I just quit at my time.*

Second, *professional incentives* were found to be threefold: 1) feeling that treatment is unsuccessful, (2) experiencing the contact with the patient as burdensome, or 3) feeling that treatment has been sufficient and (partly) successful. The latter incentive was rarely acted upon, possible because of the difficulty to judge whether treatment was ‘sufficient’ or ‘successful’, or whether the patient was ‘stable’ (see sub-section 2 on this issue). Only one professional clearly stated the need to discuss termination at the start of treatment.

Professional (Interview 9):

*‘You have to be clear, from the beginning, that one day treatment will end’*

In most cases, however, treatment (dis)continuation was ad-hoc introduced – resulting in a discussion or negotiation in which patient and professional did not always readily agree. If so, then the outcome of this negotiation was often that treatment would be continued, or that the professional would ‘discuss it in the team’.

Professional (Interview 8):

*Things are easy when someone says ‘okay, let’s quit’. But if someone does not, then you also have an important issue.*

### Getting treatment (dis)continuation on the team agenda

Getting the issue on the *professional-team agenda* is a second crucial step in the process of decision making. In all settings, final decisions were made in the multidisciplinary case conference (MCC). Professional and organisational incentives are more relevant in this stage, since patients do not generally attend MCCs in person and therefore are unable to directly influence its agenda. *Professional incentives* may be threefold. First, unsuccessful or burdensome treatments may be discussed because the professional feels stuck and hopes for good advice or help from colleagues. Second, a (partly) successful treatment may be discussed as a formality, in which professionals fulfil their duty to multidisciplinary discuss (dis)continuation – without expecting much discussion since they have already reached an agreement with the patient. Third, situations may be discussed. in which patient and professional disagree whether treatment should be terminated. There appears to be no incentive to discuss a treatment of which a professional (either or not explicitly confirmed by the patient) is certain that it should be continued. In fact, there may be disincentives to discuss anything at all, as exemplified in the citation below.

Professional [on team support when discussing patients] (Interview 7):

*The degree of support depends on the clarity of what I say to or ask of the team. Often, they all start to question me. About what happens to me, how it feels and all that.*

The perspective of being forced into an interpersonal discussion with colleagues may act as a disincentive to professionals. Only when there are sufficiently strong incentives (i.e. problems in the treatment as described above), the professional brings a patient forward. The default setting of not discussing treatment (dis)continuation was hardly questioned in teams, with one exception (see sub-section 3 on team factors). *Organisational incentives* came from the service’s or team’s policies. In our study, discussions in one team were strongly steered by guidelines on treatment duration (formulated by the service), exemplified by a typical MCC in which treatment logistics were central, and structural evaluation was standard procedure. When such organisational incentives were present, individual professionals were (socially) obliged to discuss treatment (dis)continuation – when absent, it was up to themselves.

2) **Grounds for treatment (dis)continuation**

When (dis)continuation becomes an issue in treatment (which is not always the case as we exemplified above), a number of factors may come into consideration. The precise symptomatic condition of the patient, we found, is hardly the primary concern. Although individual patient outcome data were available, they were hardly explicitly used or discussed to make decisions. Patients’ conditions were described only broadly, in terms like: ‘things go better’ or ‘she is stabile’. Given the subjective nature of such statements, they were frequently debated in team discussions. Factors taken into account are summarized in Table 2.

**Table 2:** Overview of patient-related and professional-related factors in treatment (dis)continuation decisions

Patient-related factors	Citation	Professional-related factors	Citation
*Stability* Degree to which the patient functions without crisis for at least some time, and the ability to cope effectively when problems arise or stress increases.	*‘I can stop because I feel more stable and I now have a steady relationship with someone’* (P, Interview3) *‘First work on stabilization (…). Later we can discuss termination, but first we need to relieve pressure’* (PSA, TeamMeeeting12) *‘She can come to see me if she feels that will keep her stable’* (CMHN, TeamMeeeting5)	*Trust* Degree to which the professional believes that the patient will be able to cope with future difficulties	*‘Too much hassle to terminate treatment, she will definitely be back if we do’* (CMHN, TeamMeeeting20)
*Risk* Degree to which there is: 1) danger to the patient and/or others 2) other risk: relapse, deterioration	*‘It feels sound. They can let me go now, I can go on. By myself I mean, without them’* (P, Interview11) *‘We are not done with him until the threat becomes less’* (SW, TeamMeeeting6) *‘No termination! He is a real loony, dangerous, much drug use in the past’* (SW, TeamMeeeting17)	*Fear* The feeling that ‘things may go wrong’ with the patient	*A professional (non-psychologist) on others:* *‘Psychologist are chickens, they never dare to terminate treatment’* (CMHN, Interview5)
*Motivation* Degree to which patient is willing and able to work hard to reach improvement	*‘He is now motivated but that can easily turn around, and then I don’t know if we should continue’* (PSO, TeamMeeeting15) *‘As long as he is so unmanageable we cannot discuss anything with him. We should stop’* (PSO, TeamMeeeting11)	*Reward* Degree to which the professional feels that the patient deserves another chance in treatment	*‘This lady does not fit in my treatment program but she is nice broad and I’ll give her another chance’* (CMHN, TeamMeeeting23 *‘She is avoidant, last November we agreed that she would call me but she has not. So it would be best to end care now’* (PSA, TeamMeeeting14)
*Hope* Degree to which the patient has hope that treatment will result in improvement	*‘I have to tell him that he cannot expect anything of us anymore. That the problems lie elsewhere, that he has to accept things as they are. But he does not seem open to that’* (PSO, Interview4) *‘It will not go away, but I have learnt to deal with that. In that I have succeeded’* (P, Interview11)	*Perspective* Degree to which the professional beliefs that treatment will result in improvement	*‘He does not agree with us that further treatment will not help. He really wants to quit using substances. So we are going to try another treatment and see if it works’* (physician, TeamMeeeting2)

*P: patient*

*PSO: psychologist*

*PSA: psychiatrist*

*CMHN: community mental health nurse*

*SW: social worker*

### Patient and professional factors (2a & 2b)

Patient related factors were extracted from both patients’ and professionals’ quotes. In all quotes a certain characteristic is attributed ‘objectively’ to the patient, through which respondents closely align their daily practice with professional and financial guidelines in which patient-related factors are the sole official criteria on which decisions are based. However, we found that patient factors are mirrored by professional factors that are absent from official documents. For instance, the ‘stability’ that is often desired in patient by professionals, has a professional counterpart that is much less objective. When the patient is ‘stable’, this often means that the professional has a certain amount of ‘trust’ that the patient will cope with future drawbacks, without relapsing in a crisis. The same applies to ‘risk’, that is suggested to be an objective patient factor that can reliably be assessed, yet appears closely related to professionals’ fear of ‘things going wrong’. Opposed to ‘trust’ (that the patient will cope), there may be ‘fear’ in the professional that things will go wrong – thus resulting in high ‘risk’. Without shared definitions or objective measures of ‘stability’ and ‘risk’, it is highly dependent on the individual professional whether ‘fear’ or ‘trust’ prevails – thus making the patient’s perceived condition foremost an issue of the professional’s personal assessment.

The same mirroring applies to the ‘motivation’ and ‘reward’-pair: motivation is considered a patient characteristic, that when it is high (which it preferably is), professionals tend to ‘reward’ with more treatment – as if this was a gift to offer people who strongly want treatment. When motivation is low or variable, this ‘gift of therapy’ is much less likely – professionals do not easily ‘reward’ the patient, or even push him or her away. The final pair, ‘hope’ and ‘perspective’, may reinforce the ‘rewarding process’. If a patient is optimistic about improvement, and possibly puts pressure on the professional to keep working towards it, this perspective may dominate – even when the professional believes treatment will be of no or limited benefit. As such, ‘objective’ patients characteristics are mixed up with professionals’ characteristics, and become interpersonally constructed factors in which ‘objectivity’ is hard to maintain.

### Organisational and environmental factors (2c & 2d)

Organisational and environmental factors also are strongly intertwined (Table 3). The *internal capacity* of the mental health service may influence treatment (dis)continuation. For example: when there are many people on the wait list, the internal capacity is limited. Yet, if a new treatment program is opened, or the team’s capacity is extended, the internal capacity is relatively large. This likewise applies to treatment options outside the own service, and thus *external capacity* in the local or regional network may shape treatment (dis)continuation – if available, patients may be referred elsewhere. *Financial incentives* influence this process in covert ways: since mental health services do not receive reimbursement per professional, but only per patient contact, it is highly unattractive to have ‘too few patients’. Professionals, knowing that too few contacts will make themselves and the service vulnerable, may make treatment (dis)continuation decisions based on financial incentives. For instance: by terminating treatment of patients that often do not show up or by continuing treatment of compliant, loyal and ‘easy’ patients. The final two factors from our overview (Table 3) are *resistance* and *support.* Professionals may want to terminate treatment when they consider the patient sufficiently stable and not presenting risk (see Table 2). Yet if others disagree, resistance (possible followed by hassle, complaints, and legal action) may prevent termination. This interdependent principle also works the other way around: if social support for the patient is abundant, professionals more easily decide to discontinue treatment.

**Table 3:** Overview of organisation-related and environment-related factors in treatment (dis)continuation decisions^a^

Organisation-related factors	Citation	Environment-related factors	Citation
*Internal capacity* The degree to which treatment options are available within the service	*‘She has not shown up in our program, the DRG has ended and we are not going to open a new one. So let’s quit’* (PSO, TeamMeeeting4)	*External capacity* The degree to which treatment options are available outside the service	*‘This is highly complex care. If the patient has five contacts per year we may keep him stable. This does not have to be in our service – if the primary care physician has a mental health nurse it may be done there’* (PSA, TeamMeeeting7)
*Financial incentives* The degree to which financial reimbursement of services influences decisions	*‘Limitations play no role. If there is still is a need for treatment, another DRG* [financial treatment episode] *is started’* (CMHN, Interview8)		
		*Support* The degree to which the patient has social support, or is able to organize practical support	*‘The biggest problem was her housing. Once she got another house, things were already a lot better. When her financial situation got better also, we were able to terminate treatment’* (SW, TeamMeeeting8)
		*Resistance* The degree to which others (e.g. family, neighbours, other institutions) are willing to care for the patient	*‘This is an annoying guy but if we terminate treatment there will be trouble that will reflect on us’* (CMHN, TeamMeeeting11)

^a^Please note that not all cells are filled, since in the analysis not every factor was found to apply to both the organisation and the environment

### The influence of substantial factors on decision making

We identified a number of factors implicitly influencing treatment (dis)continuation. However they hardly explicitly informed the decision making process. There were no clear indicators of ‘stability’, or ‘risk’ and therefore ‘trust’ and ‘fear’ become important factors influencing the patient-related criteria, and thus possible decisions. Likewise, external guidelines on what is sufficient treatment (provided by the organisation), or a socially acceptable outcome (provided by the environment) were absent, and therefore decisions appeared mainly to be based on supply/demand-logistics or societal response.

3) **Team-related factors on which team decisions may be made**

In the fourth and final sub-section, we will show how decisions are made in teams, but first we need to describe team factors that may influence team decision making (Table 4). Structural elements, like frequency and duration, varied somewhat among teams but did not appear relevant to the decision making process. For example, in a longer meeting more patients could be discussed, but decision making happened relatively independent of available time. Cultural factors were of larger importance, therefore we discuss these in detail below.

**Table 4:** Team related factors: structural and cultural elements of team meetings

Team	1	2	3	4
Structural elements				
Frequency	weekly	weekly	Weekly	weekly
Duration	1 h	1.5 h	2 × 1 hour	1.5 h
Cultural elements				
Type of meeting	‘structured’	‘unstructured’	‘unstructured’	‘unstructured’
Urgent cases	last	first	first	last
Type of chairing	technical	substantial	substantial	substantial
Presence of team members	in-out	all	all (in theory), few (in reality)	all
Atmosphere	critical, controlling, case-focussed	supportive, permissive, case-focussed	supportive, permissive, team-focussed	supportive, permissive, case-focussed

#### Type of meeting

Three of the four MCCs were identified as ‘unstructured’, meaning that there were no pre-set time limits, or predetermined number of cases. As such, one case could be discussed at length, while another case received little or no attention. This was relevant since treatment (dis)continuation may thus not be discussed, in spite of the professional putting it on the agenda. A central issue in all teams was the division of scarce time between ‘regular’ and ‘urgent’ patients. The first group had to be discussed every 6 months, according to protocol, the latter group required immediate attention due to a variety of reasons (e.g. a patient’s crisis, a deadline for referral, or some other issue). Irrespective of whether urgent patients were discussed first or last, their ‘urgency’ overshadowed the discussion of regular patients. In all teams, discussion of regular patients was often postponed to the next meeting. This process resulted in structurally devoting more time to urgent patients (for whom treatment termination is not an option due to their urgency), and less time to regular patients (for whom treatment termination may be an option). The one team in which meetings were more structured did in fact discuss urgent patients last, and succeeded more often in discussing treatment (dis)continuation of regular patients.

#### Type of chairing

The chairperson had an important role in discussions and decision making. In three out of four teams the chairperson was the team leader, in one it was a (monthly) rotating team member. Differences were substantial: some chairpersons confined themselves to ‘technical’ chairing, some participated actively in decision-making. The way professionals introduced and discussed patient situations varied across teams. However, a lengthy introduction was default and often professionals engaged in long conversations in which the outcome of the discussion moved out of focus. Also, a clear question for the team was hardly verbalized. Sometimes, (technical) chairs intervened to structure these discussions, at times resulting in clearer decision making – but not in all cases.

#### Presence of team members

The degree to which professionals were present varied. In one team, professionals were present only when ‘their’ patients were discussed while solely the chairperson and the team’s psychiatrist were constantly present. In another team, the intention was to have all members present all the time, yet professionals ‘on call’ walked in and out repeatedly. In such an in-and-out structure, discussions could be repetitive and decision making was often postponed due to the absence of one or more team members. Thus, while planned ‘absence’ appeared to be helpful, ad-hoc absence was not helpful to discussions on treatment (dis)continuation.

#### Atmosphere

The last element that influenced content and form of discussions, here called atmosphere, was closely connected to group dynamics. The interpersonal relationships within the team interacted with all of the aforementioned cultural variables. Some team members were more influential in discussions, regardless of their formal position in the team (e.g. as responsible clinician, chair, or team leader). The atmosphere in some teams appeared more open and warm, with mutual interest in one another, including personal issues. Other teams were more strict and business-like, with little room for personal contact. A business-like atmosphere, however, did not necessarily result in a more focussed discussion of treatment (dis)continuation.

4) **Team decision making on treatment (dis)continuation**

The team decision making process may be seen as the ultimate outcome of the process of getting to decision making (sub-section 1), substantial factors (sub-section 2), and team factors (sub-section 3). Here, we divide decision making in *process* and *outcome*.

### Process of team decision making

Earlier we described the somewhat chaotic way patient cases are introduced, and how urgent cases may overshadow regular cases. This unstructured way of discussing was reinforced by the habit of team members to frequently ask for clarification. Their lack of familiarity with the patient apparently urged them to request more details to understand the situation. This focus on detail often prevented an outcome-focused discussion of the situation – moving decision making out of sight. The degree to which a focused (dis)continuation discussion, instead of just a full patient history taking, took place, depended on some factors. The preparation of team members: the more they were informed beforehand, the more substantial the discussion. The familiarity of professionals with the patient: the more familiar, the more substantial. And the number of professionals familiar with the patient: the more professionals familiar, the more substantial.

In none of the teams decisions were made by the entire group, this mostly happened in groups of two to four professionals. Formal decision rules, e.g. that at least a certain number of team members had to consent on a decision, were not observed or expressed in any team. Clear decisions on (dis)continuation were rare, often there was no unequivocal conclusion. A decision was most likely when the introducing professional had a strong conviction that treatment should be terminated. However, even when a professional introduced a clear intention, other team members moved the discussion in various directions (example 1).

**Example 1: professional wants to terminate treatment, yet the patient does not**

(Team meeting 11)

*PSO (psychologist): This lady is addicted to mental health services*

*PSA (psychiatrist): Let’s shake her hand, tell her how well she is doing, and terminate treatment as soon as possible*

*PSO: Yes, indeed*

*CMHN (community mental health nurse) suggests: She can come see* me *if she believes that will keep her stable*

*PSO2: Isn’t this a good fit for a social worker?*

*PSO: All this lady does, is consume care*

*PSA: Her demand ‘stay with me’ seems real, can we really terminate? Maybe a careful referral to social work?*

*PSO: I don’t know, I think we should end specialized services*

*PSA: Why not consult with her primary care physician? If they have a CMHN in their practice, she could go there for a couple of times a year.*

*PSO: I do not believe she will accept that, she has many problems but does not want to switch professionals again*

*PSA: Please check if there is a CMHN in the general practice, if not she remains with our own CMHN*

In this example, we see that the psychologist’s perspective (treatment should be terminated) is initially backed up by the team’s psychiatrist, until the option of another type of care – by a community mental health nurse (CMHN) or social worker – is introduced. Then, the psychiatrist starts to express doubts, introduces a CMHN as an option, and concludes that if no such care is available the patient *should* stay in specialized services – albeit with another professional than the psychologist who introduced the case. This example is prototypical for many multidisciplinary meetings: individual and organizational disincentives to terminate treatment are collectively reinforced. Almost always, one or more of the present professionals hopes for a positive outcome, or has a suggestion for alternative treatment, regardless of previous attempts. Given the fact that meetings are often unstructured, professionals that verbalize ‘hope for improvement’ may have an advantage over those that verbalize ‘enough is enough’ – which is more negative and therefore less socially accepted. In almost all meetings, decisions were based on the loudest majority in the team, as well as on other factors unrelated to the patient’s condition (e.g. the internal or external treatment capacity).

Even when the discussion was limited to two persons (example 2), it appeared to be difficult to refrain from alternative options that would keep a patient in treatment. In fact, the absence of another team member involved in treatment, may prevent the decision to terminate.

**Example 2: professional introduces patient to discuss uncertainty about (dis)continuation**

*CMHN: I should make a new appointment with him but I don’t know if it is still necessary*

*PSA: Maybe treatment can be terminated?*

*CMHN: I feel that now he has this structure in life, he may possibly start going to school. I have suggested this and he liked the idea, he likes to be in charge again.*

*PSA: We need to check this with his doctor here* [a psychiatrist-trainee, not present at the meeting]

*CMHN: I am not sure about his medication?* [after checking this in the file] *Medication is already prescribed by his primary care physician, not by our doctor. Also he now knows what his psychosocial problems are, and since…*

*PSA: If he does not want to terminate treatment, we can still refer him to the CMHN in the primary care practice? Or refer elsewhere?*

*CMHN: I feel he is ready to terminate treatment*

*PSA: Will you discuss it with him? He can see our doctor once more for his meds, although I am not sure if that is even necessary*

*CMHN: I strongly suggest to terminate treatment now*

*PSA: Or could maybe you and the doctor do it altogether? That would be most…*

*CMHN: That will push it forward for another four weeks* [doctor is currently unavailable]

*PSA: But are you able to meet him* [patient] *before his appointment with her* [the doctor]*?*

*CMHN: Yes*

*PSA: Our doctor is on holiday, otherwise she could have joined you*

*CMHN: She will most certainly agree*

*PSA: So, a final meeting, followed by a discharge letter to the primary care physician*

In example 2, larger group dynamics were limited but there seemed a reluctance in the team’s psychiatrist to terminate treatment during the meeting. While the CMHN became more convinced of the need to terminate treatment, the psychiatrist opted for a termination under the condition that the team’s doctor would see the patient one more time. Thus, while the CMHN started hesitatingly (‘I don’t know’) and the psychiatrist started rather firmly (‘Maybe treatment can be terminated’), they ended in opposite positions with the CMHN convinced (‘I strongly suggest to terminate treatment now’) and the psychiatrist in doubt.

### Outcome of team decision making

In both discussions, as in many others observed, the substantive criteria (Table 2) were not explicitly used. Instead, the environmental criteria (Table 3), specifically the availability of treatment by another professional, were dominant. There appeared an inclination towards treatment continuation – even when the treating professional explicitly argued to discontinue. Interestingly, this inclination could not easily be ascribed to professionals being fundamentally unwilling to discontinue treatment since – as exemplified in both examples – professionals rapidly changed their perspective during a team discussion.

Instead, decision making is an interpersonal negotiation: different perspectives are discussed and professionals may change their perspective depending on who are present during the meeting, the respective positions of these professionals in the team, and the group coalitions made. Regardless, a decision easily gravitates towards treatment continuation. Continuation is default – discontinuation requires a strong determination. Our main finding, and core category of the Grounded Theory-process, is that treatment (dis)continuation depends more on interpersonal negotiation than on objective weighing of criteria, with treatment continuation as the default outcome. Some criteria are available, yet they are poorly measured and hardly explicitly used. More than one negotiation needs to be navigated before (dis)continuation is properly discussed in the MCC – once that happens the outcome appears to be rather random.

## Discussion

Our main research question was on what grounds, and by which processes, multidisciplinary outpatient teams, individual professionals, and clients decide to (dis)continue long-term psychosocial treatment. This study yielded many variables shaping decision making on treatment (dis)continuation. However, our main finding is that treatment (dis)continuation depends more on interpersonal negotiation than on objective appraisal of criteria, with treatment continuation as the default outcome. This negotiation takes place on the patient-professional level, on the agenda setting of the professional-team level, and in the eventual professional-team discussion.

### Results

Looking at our results, we cannot but conclude that discussing psychosocial treatment (dis)continuation in secondary mental health services is a complex, unstructured activity. Professionals find it difficult to discuss with patients, unless the patient brings it up. Professionals find it difficult to discuss it in their teams, and teams find it difficult to discuss it in a structured way, to reach a decision – and if they decide at all – to discontinue treatment. We discuss four potential explanations.

*First*, in long-term psychosocial treatment there are no golden rules on either outcome or process. If and when a patient is sufficiently improved or recovered (*outcome*) is unclear since often symptoms remain. Yet, even when a person is ‘objectively’ recovered, this does not mean that he or she feels as such [28, 29] ‘Recovery’ is a very personal and multi-interpretable issue that (thus far) cannot be objectively captured. If and when sufficient treatment has been offered (*process*) is also unclear since there is no prescription on treatment duration. It is open to debate whether ‘hard’ measurement of outcome is impossible in long-term psychosocial treatment. Although not used in the services in our sample, generic outcome measurements (e.g. Outcome Rating Scale [30]) have shown to be useful in measuring progress – and possible in planning (dis)continuation.

*Second*, when objectivity is absent and much is uncertain, professionals rely on experience (their own clinical judgment) to make decisions. Professional clinical judgment, however, is potentially unreliable, especially regarding risk assessment [31]. A cognitive professional bias may be present (the ‘insider-perspective’ [32]), by which the patient appears very ill to the professional during sessions, but in fact may function relatively well outside treatment– unknown to the professional. Studies found that professionals overestimate risk and underestimate strengths in patients [33, 34]. Low-frequent but high-impact incidents with patients, receiving high media-coverage, may reinforce risk-averseness. In general, in the absence of ‘objective’ decision making ‘technology’, professionals may (unconsciously) use heuristics to make decisions – consisting of core beliefs about people with mental illness, professional responsibility, and notions about care and risk. We have touched on some of these (e.g. trust and fear; see Table 2) but more may be at play [35].

*Third*, another strategy to deal with uncertainty is to rely on consensus. Consensus may be sought with the patient or within the team. When consensus with the patient is found – implicitly or explicitly – there is no problem: both want to end, or both want to continue treatment. When this consensus is however not found, the professional may try to find consensus in the team. Yet, as we have seen, professionals do hardly ever reach consensus, even when strong argument are made. Underlying beliefs and possible risk-averseness observed in individual professionals, may also apply to teams during discussions. Therefore it is unlikely to reach consensus on changing standing practice – and treatment continuation thus is the default outcome.

*Fourth*, the treatment environment hardly favours changing default settings. Patients’ complaints about – in their view – prematurely terminated treatments are undesirable from the organisation’s perspective. Also, a rapid turnover of patients due to short-term treatment is more labour-intensive (more assessing and reporting is required) and financially risky (empty slots in the professional’s agenda are costly). In general, the multidisciplinary decisional structure of many organisations does not support clear decision making (process) and changing the status quo (outcome) [36, 37].

### Strengths and weaknesses

This study is one of few empirical studies into decision making on long-term psychosocial treatment (dis)continuation so far. Strong points are the variation in mental health services (three), teams (four), and professional backgrounds (> 10). A substantial number of MCC’s was attended (29), and participants were interviewed (12). However, few clients were eligible – which may have obscured full understanding of the patient-professional interaction. Also, one of the so-called ‘patient-related factors’ (i.e. motivation) in Table 2 is not as such described by patients, but only by professionals.

In terms of qualitative research rigour [38], we focused on credibility, transferability, dependability, and confirmability. By interviewing multiple key informants, and using both individuals and teams as data sources, data triangulation was applied. Investigator triangulation was applied in the research team, method triangulation was applied by using both observations and interviews. Debriefing with peers took place within the research group, and intermediate results were discussed in two expert workshops – with professionals and patients outside the study. Member checks were performed to ensure the researcher’s proper understanding of answers, by giving summaries during interviews, and by requesting additional information or explanation from team members following team observations. Careful selection of the research setting took place, focusing on teams in which the process of decision making was both relevant and frequent. Stability of the data over conditions was assessed by using four different teams/settings as data sources. Although differences between teams were noted, the main process described was found in all teams. A detailed log book, consisting of memo’s about data collection, analysis, and interpretation, was kept.

The generalizability of the results across health care systems outside of the Netherlands is an open question. While decision making on psychosocial treatment (dis)continuation appears difficult in many systems and countries, local financial constraints may guide decision making in different ways than encountered in this Netherlands-based study. However, in nations with universal health care coverage, e.g. the UK’s National Health Services, no guidelines on treatment discontinuation exist and in times of larger budgets, community mental health treatment may continue until the person is referred to another team, or back to primary care.

### Implications for practice

Although long-term psychosocial treatment is not the standard for most people using mental health services, it is for many – and often its termination is complicated. Professionals’ reliance on ‘objective’ outcomes and professional consensus appear unwarranted – we found evidence only for an interpersonal negotiation, or construction, of decision making. An early dialogue between patient and professional on goal, course, and termination of psychosocial treatment may be very helpful to improve mutual awareness of the process-like character of treatment, to keep overly high expectations at bay, and to foster a mutual responsibility for process and outcome. Such procedures are effective in facilitating treatment termination in situation of limited time (brief therapy) or money (health care systems without long-term coverage), and may also be helpful in situations in which psychosocial treatment should or can be longer. When patient and professional make the treatment process a truly mutual, interpersonal undertaking, decision making may also be more mutual - and less become a distant, objectified, and consensus-based team task.

## Conclusions

Clear decision making, and terminating treatment when appropriate, is systematically hampered within mental health services. Since treatment continuation is the more easy default option, treatment termination requires a skilful and determined navigation of at least three interpersonal negotiations. Given services’ scarce means, people’s large demands for help, and patients’ unused potential autonomy, it may be beneficial to invest in decision making skills and procedures – both human and economic profits may be substantial.

## Abbreviations

CMHN: Community Mental Health Nurse
DRG: Diagnosis Related Groups
MCC: Multidisciplinary Case Conference
PSA: Psychiatrist
PSO: Psychologist
